# A database for using machine learning and data mining techniques for coronary artery disease diagnosis

**DOI:** 10.1038/s41597-019-0206-3

**Published:** 2019-10-23

**Authors:** R. Alizadehsani, M. Roshanzamir, M. Abdar, A. Beykikhoshk, A. Khosravi, M. Panahiazar, A. Koohestani, F. Khozeimeh, S. Nahavandi, N. Sarrafzadegan

**Affiliations:** 1 Institute for Intelligent Systems Research and Innovation, Deakin University, Geelong, VIC 3216, Australia; 2 Department of Electrical and Computer Engineering, Isfahan University of Technology, Isfahan, 84156-83111 Iran; 3 Département d’informatique, Université du Québec à Montréal, Montréal, Québec Canada; 4 Applied Artificial Intelligence Institute, Deakin University, Geelong, Australia; 5 University of California San Francisco, San Francisco, CA USA; 6 Mashhad University of Medical Science, Mashhad, Iran; 7 Isfahan Cardiovascular Research Center, Cardiovascular Research Institute, Isfahan University of Medical Sciences, Isfahan, Iran; 8 School of Population and Public Health, Faculty of Medicine, University of British Columbia, Vancouver, British Columbia Canada

**Keywords:** Cardiovascular diseases, Data integration, Data mining

## Abstract

We present the coronary artery disease (CAD) database, a comprehensive resource, comprising 126 papers and 68 datasets relevant to CAD diagnosis, extracted from the scientific literature from 1992 and 2018. These data were collected to help advance research on CAD-related machine learning and data mining algorithms, and hopefully to ultimately advance clinical diagnosis and early treatment. To aid users, we have also built a web application that presents the database through various reports.

## Abstract (summary)

**Table:** 

Measurement(s)	coronary artery disease
Technology Type(s)	digital curation
Factor Type(s)	year • disease
Sample Characteristic - Organism	Homo sapiens

Machine-accessible metadata file describing the reported data: 10.6084/m9.figshare.9825680

## Background & Summary

According to the World Health Organization (WHO) available in http://www.who.int/news-room/fact, cardiovascular diseases (CVDs) are the major reason for death worldwide. CVDs include different diseases related to heart and blood vessels, such as coronary heart disease (CHD), cerebrovascular disease, and rheumatic heart disease (RHD) among others. According to the latest WHO report available at http://www.who.int/news-room/fact and http://www.who.int/cardiovascular_diseases/en/, more than 17.7 million people are estimated to have died in 2015 due to having CVDs, accounting for 31% of all deaths globally. It also estimated that approximately 7.4 million died due to CHD, which is also called coronary artery disease (CAD)^1–3^. In other words, it can be argued that CVDs - in particular, CAD - are among the deadliest diseases in both developed and developing countries and paying attention to them is vital and indispensable.

Although the CAD mortality rate is high, the chance of survival is higher if the diagnosis is made early enough. Therefore, scientists have devised predictive models to identify high-risk patients. Recently, machine learning (ML) and data mining (DM) approaches have become more popular to construct models not only for the early diagnosis of CAD^4–11^ but also for other fatal diseases^12–17^ such as cancer^18–24^. These techniques reveal the hidden structures that help to achieve a quicker diagnosis among the large amount of medical data^25^. Indeed, this is a semi-automated approach for finding patterns in data^26^.

Although there are some datasets for various diseases^27–40^, there are no comprehensive benchmarks publicly available to summarize the research and conclusions on CAD diagnosis. As a result, the studies in this field are not well organized. One can argue that a solution to this problem is creating a database of all studies to collect their related information. Using this database, researchers can explore the latest work in the field and stay informed about the new methods proposed and the results achieved. Therefore, this research attempts to provide a comprehensive dataset of the related works at the intersection of ML/DM and heart disease detection as a bridge for further research in the future. The impact of CAD disease on our daily lives and the popularity of ML/DM motivated us to create such a database. To the best of our knowledge, this is the first database that covers most relevant datasets as well as the related outcomes obtained by ML algorithms. It is a key point to recognize other modifications on ML/DM techniques that are relevant to CAD disease progression and development.

This database provides comprehensive and fundamental information on early detection of CAD disease in order to illuminate the patterns and processes that are used in ML/DM approaches. For instance, Alizadehsani *et al*.^3^ used an SVM to classify patients with CAD from healthy individuals. Their model had an accuracy of 95% and revealed that apart from typical chest pain, regional wall motion abnormality and ejection fraction (echocardiographic features), age, and hypertension have the highest importance in CAD diagnosis. Therefore, ML/DM techniques will be fruitful to biologists, computer scientists, healthcare researchers, and physicians who are experts in the CAD area.

The advantages of using ML/DM methods for CAD diagnosis can be summarized as follow^3^:

- It may result in early detection that leads to a decrease in mortality rate.
- ML/DM can provide a priori probability of disease and use this probability to selectively target patients for angiography. This can save in cost and time for other patients. The side effects of angiography are also eliminated for them.
- Using ML/DM can extract hidden patterns in the collected data. This may lead to finding new methods for early detection in many diseases like CAD.

Although ML/DM techniques have many advantages, they are not perfect methods. The following factors limit their abilities in some directions.

- According to no-free-lunch theorem^41^, different ML/DM algorithms are suitable for their own particular problems. One algorithm may work well on a specific dataset while it cannot show a good performance on some others. So, selecting a suitable algorithm for a specific dataset is a big challenge in bioinformatics. Consequently, selecting good feature selection or classification algorithms is also a big challenge in this field.
- ML/DM algorithms commonly need massive datasets to be trained. These datasets must be inclusive and unbiased with high quality. Datasets also need time to be collected^42^.
- ML/DM algorithms need time to be trained and tested enough to be able to generate results with high confidence. These algorithms need a lot of resources and equipment^43^.
- ML/DM algorithms face the verification problem. It is difficult to prove that the prediction made by them work correctly for all scenarios^43^.
- The correct interpretation of the generated results by ML/DM algorithms is another challenge that we are faced with^42^.
- Another disadvantage of ML/DM algorithms is their high error-susceptibility. If they are trained with biased or incorrect data, they end up with imprecise outputs. This may lead to a chain of errors that mislead treatment methods. When these errors get noticed, it takes some times to diagnose the source of these errors and even needs more time to correct them^44^.

The benefits of using our collected dataset can be listed as follows:

- The researchers can access useful information easily and quickly to the results of much state-of-the-art research in this field. For example, important features on CAD in each country, comparing the performance of different research, features which were used in each research, and many other useful information that can be extracted from this dataset. Consequently, researchers can find the fields that there are fewer works on them. It also prevents researchers from doing repeated works.
- This dataset facilitates the review step of the researchers. They can do their research quickly and with more quality. Meanwhile, the referees can also use it to check the novelty of new proposed methods and have quick access to the important properties of the articles. Using this dataset, top rank researchers and better algorithms and journals with more published works in this field can be found easily. It can also be extended for diagnosing other diseases especially more common ones such as diabetes, cancers, and hepatitis.

However, unfortunately, this dataset suffers from some disadvantages. Currently, updating the dataset is done manually. For example, finding new papers, extracting their properties and adding them to the dataset are done manually.

Meanwhile, one of the most important weaknesses of the research we investigated is the size of the used datasets. Unfortunately, almost all of the researchers do not use big datasets because collecting too many records needs a lot of time and cost. If we want to have extremely high confidence results, we need more than one million records. Projects like Electronic Health Records (EHR)^45^ can help to achieve this goal. In EHR project, information about patients is saved electronically in a digital format. It can be shared across different health care centers to ease the treatment process. EHR almost includes all necessary records of patients like their medical history, drugs that are used, their procedures, vitals and their allergies, and laboratory test results. This mechanism has improved the quality of care. By increasing the samples in EHR, the quality of cure methods will be improved definitely. Meanwhile, it can reduce the risk of data replication as there is only one medical file for each patient which is commonly completely updated file. As all the information about the patients is saved in a digital searchable file, EHRs are more effective for extracting information for treatment methods. Meanwhile, population-based methods can also be applied more easily by widespread adaptation of EHR.

States of the art methods like deep learning can also benefit from EHR because deep learning needs much data for learning and these data are collected in EHR. Deep learning^46^ is a part of machine learning algorithms based on artificial neural networks. Nowadays, this method shows significant ability in solving machine learning problems. It is inspired by distributed processing of biological systems. Because of its ability in the learning process, nowadays, it is used in various learning fields like machine vision, image processing, and bioinformatics.

Deep Survival Analysis^47^ is a hierarchical generative approach using EHR for survival analyzing. It handles characteristics of EHR data and for an event of interest, it enables accurate risk scores. Traditional survival analysis^48,49^ suffers from some weaknesses. For example, high dimension and very sparse data of EHR are one of these weaknesses that makes using traditional model difficult. Deep Survival Analysis differs from traditional survival methods. In this method, all observations are modeled jointly and conditioned on the rich latent structure.

As a clinical implication of this research, physicians can use this dataset to select more effective features in CAD diagnosis according to the region they are living in. This can increase the accuracy of their diagnosis and help the early treatment of patients. Meanwhile, as it was mentioned, it can also reduce the usage rate of angiography for suspicious patients and avoiding the side effect of the unnecessary procedure. More importantly, our system will work as a recommendation engine for clinicians to help them in decision making for specific treatment for a specific group of patients with similar characteristics toward personalized medicine.

## Methods

For the first time, we designed and implemented a complete dataset about the research in CAD diagnosis field. It is an important field and many researchers work on it. So, accessing to a complete resource of the research in this field can help researchers improve their work more quickly and precisely. Meanwhile, this dataset includes some useful utilities for extracting information from the data saved in it. These utilities are accessible in www.cadataset.com. Using this dataset, some new information can be extracted. For example, a physician can find what features are more important in CAD diagnosis in different regions.

This study concentrates on recent papers from 1992 to 2018 that are related to CAD diagnosis using ML/DM techniques. For the sake of completeness, we used Google Scholar to find the most related articles. The database includes information such as authors, publisher, title of the paper, country (of publication or where the research was conducted), methods that are applied, evaluation metrics, type of diseases, features that are used, journal/conference, and the most important features used in their analysis (e.g., Alizadehsani *et al*., Elsevier, a data mining approach for diagnosis of coronary artery disease, Iran, [SVM, Naïve Bayes], [Accuracy, Sensitivity, Specificity], CAD, 55 features, computer methods and programs in biomedicine, 36 features).

As many CAD-related articles are published every year, we built the dataset such that it can be easily updated. Using this database, for each paper published in the field, one can determine in which countries the data are collected and what features have been reported to be of importance. In addition, features not considered in each study are also determined, allowing researchers to examine those features in the future. It also reveals which authors have more influence in the field, so others can use their experiences. The journals that have published the most articles in this field are determined to allow researchers to decide on where to publish their new articles. All of the algorithms that have been used to date and the accuracy that they have achieved are identified so that researchers can choose the algorithms that have not yet been used and compare them with previous results. The articles that have the most citations have been identified so that researchers can use their ideas. The publication houses that have published the most articles in this field are identified. The datasets and the feature categories that achieved the most accuracy are determined to help researchers in feature selection. The feature selection algorithms that researchers have used have been identified to help new researchers choose the best method. The articles that have achieved the most accuracy are specified to help researchers decide on which features and methods have better results.

As the future work, there are multiple issues for improvement of mechanisms used for collecting and management of our dataset. They are summarized as follows:

- There are no published data for most countries in Europe, Africa, Australia, and South America. This lack of information is important as regional and racial differences may affect the way CAD is detected and treated. Thus, we recommend collecting CAD data and constructing databases from various continents and countries.
- Most of the investigated datasets have a limited number of features. This severely limits the final results since the number of both samples and features can affect the performance of ML techniques. Hence, we will construct CAD databases with more features.
- The median sample size for the CAD datasets that were investigated in research in this field is less than 500. The larger the number of samples is, the more significant are the statistical results. To ensure reliability and trustworthiness, a model should be developed and tested using at least one million samples^50^.
- Another problem with previous studies is the way the data were collected. Since the datasets differ in terms of the number of samples and features, it is not easy to compare ML techniques in terms of performance. In other words, the results obtained in various studies are comparable only if the data are the same. This dataset can help researchers to select features that make their research comparable with others.
- As it was mentioned, currently, updating our dataset is done manually. Improving the tools which now is used to manipulate our dataset is necessary. This tool must be able to update the dataset automatically.

### Database structure

The database designed in this research includes 15 tables shown in Tables 1–15. These tables include the following information:

- the field name,
- whether it is a primary key (P.K.) or a part of it,
- if it is a foreign key (F. K.), and if yes, to which table it refers,
- and a brief description of that field.

Table 1 lists the journals and conferences in which the investigated papers were published. In Table 2, the authors of the investigated papers are listed. Commonly in each paper, there are one or more datasets to which the proposed algorithms were applied. These datasets are listed in Table 3. Currently, we investigated only four heart diseases that are listed in Table 4. They are CAD and stenosis of LAD, LCX, and RCA. This list, however, is extendable to other diseases in the future. The features investigated in the articles are listed in Table 5, and the list of methods used for diagnosis is shown in Table 6. In most of the papers, the researchers selected a subset of features in the investigated datasets. The feature selection algorithms are listed in Table 7. Table 8 is dedicated to the characteristics of research papers but not the review papers in the field. The characteristics of review papers are shown in Table 9. Table 10 shows which method was applied on a specific disease in a dataset in a specific paper. The results of applying this method are also reported. Table 11 shows the features of each dataset. Table 12 indicates the authors of each research paper, while Table 13 indicates the authors of review papers. Since we separated the tables of research papers and review papers, we did the same for their authors as well. The research papers and review papers have different fields to report on. Therefore, we used different tables to save their details. To specify the feature selection algorithm that was used in each paper, Table 14 is designed. Finally, in Table 15, the rank that was assigned to each selected feature was reported.

**Table 1:** The fields of “Journals/Conferences” table, their properties and descriptions.

Field name	P. K.	F. K from Table (Field)	Description
Journal/ConferenceID	✓		The ID defined for each journal or conference
Journal/ConferenceType			This field indicates if this record is a journal or conference
Journal/ConferenceName			The name of the journal or conference
Publisher			The publisher of journal or conference

**Table 2:** The fields of “Authors” table, their properties and descriptions.

Field name	P. K.	F. K. from Table	Description
AuthorID	✓		The ID defined for each author
AuthorName			The name of the author

**Table 3:** The fields of “Dataset” table, their properties and descriptions.

Field name	P. K.	F. K from Table (Field)	Description
DatasetID	✓		The ID defined for each dataset
DatasetName			The name of the dataset
DatasetSampleSize			Number of records in each dataset
Country			The name of the country where the dataset was collected

**Table 4:** The fields of “Disease” table, their properties and descriptions.

Field name	P. K.	F. K from Table (Field)	Description
DiseaseID	✓		The ID defined for each disease
DiseaseName			The name of the disease (for now they are only CAD or stenosis of LAD, LCX or RCA)

**Table 5:** The fields of “Features” table, their properties and descriptions.

Field name	P. K.	F. K. from Table	Description
FeatureID	✓		The ID defined for each feature
FeatureName			The name of each feature
Abbreviation			Abbreviation of each feature (if it exists and is used commonly)
FeatureCategory			The category that the feature belongs to

**Table 6:** The fields of “Methods” table, their properties and descriptions.

Field name	P. K.	F. K. from Table	Description
MethodID	✓		The ID defined for each method
MethodName			The name of the machine learning method that was used in each study
MethodCategory			The category that this method belongs to

**Table 7:** The fields of “Feature Selection Algorithms” table, their properties and descriptions.

Field name	P. K.	F. K from Table (Field)	Description
FeatureSelectionID	✓		The ID defined for the feature selection algorithm
FeatureSelectionName			The name of the feature selection algorithm

**Table 8:** The fields of “Papers” table, their properties and descriptions.

Field name	P. K.	F. K from Table (Field)	Definition
PaperID	✓		The ID defined for each paper
PaperName			Title of paper
FirstAuthorID		Authors (AuthorID)	The ID of the first author
Year			The year this research has been published
Journal/ConferenceID		JournalsConferences (Journal/ConferenceID)	The ID of the journal or conference that this research has been published in
Train-Test Separation Method			Which method is used for the train and test separation method
ShortDescriptionAboutMainMethod			A short description of the main method
ConclusionsReportedByAuthors			A short description of the conclusion
NumberOfCitation			Number of citations of this research

**Table 9:** The fields of “Review Articles” table, their properties and descriptions.

Field name	P. K.	F. K from Table (Field)	Description
PaperID	✓		The ID defined for each paper
PaperName			Title of paper
FirstAuthorID		Authors (AuthorID)	The ID of the first author
Year			The year this research has been published
Journal/ConferenceID		JournalsConferences (Journal/ConferenceID)	The ID of the journal or conference that this research has been published in
InvestigatedResearchFrom			The year the investigation begins
InvestigatedResearchTo			The year the investigation ends
Number of investigated papers			Number of papers investigated in each review paper
Number of citations			Number of citations of each review paper
NotableConclusion			Notable conclusion of each research

**Table 10:** The fields of “Papers_ Datasets_Diseases_Methods” table, their properties and descriptions.

Field name	P. K.	F. K from Table (Field)	Description
PaperID	✓	Papers (PaperID)	The ID defined for each paper
DatasetID	✓	Datasets (DatasetID)	The ID defined for each dataset
DiseaseID	✓	Disease (DiseaseID)	The ID defined for each disease
MethodID	✓	Methods (MethodID)	The ID defined for each method
IsMainMethod			If this method is the main method (the method with the highest performance) of the research or not?
Accuracy%			The reported accuracy
Sensitivity(Recall)%			The reported sensitivity
Specificity%			The reported specificity
F-Measure%			The reported F-Measure
AUC			The reported AUC
Precision%			The reported precision

**Table 11:** The fields of “Datasets_Features” table, their properties and descriptions.

Field name	P. K.	F. K from Table (Field)	Description
DatasetID	✓	Datasets (DatasetID)	The ID defined for each dataset
FeatureID	✓	Features (FeatureID)	The ID defined for each feature

**Table 12:** The fields of “Papers_Authors” table, their properties and descriptions.

Field name	P. K.	F. K from Table (Field)	Description
PaperID	✓	Papers (PaperID)	The ID defined for each paper
AuthorID	✓	Authors (AuthorID)	The ID defined for each author

**Table 13:** The fields of “Papers_Authors (review articles)” table, their properties, and descriptions.

Field name	P. K.	F. K from Table (Field)	Description
PaperID	✓		The ID defined for each paper
AuthorID	✓		The ID defined for each author

**Table 14:** The fields of “Papers_FeatureSelectionAlgorithms” table, their properties, and descriptions.

Field name	P. K.	F. K from Table (Field)	Description
paperID	✓	Papers (PaperID)	The ID defined for each paper
FeatureSelectionID	✓	FeatureSelectionAlgorithms (FeatureSelectionID)	The ID defined for each feature selection algorithm

**Table 15:** The fields of “Papers_ImportantFeatures” table, their properties and descriptions.

Field name	P. K.	F. K from Table (Field)	Description
PaperID	✓	Papers (PaperID)	The ID defined for each paper
DiseaseID	✓	Diseases (DiseaseID)	The ID defined for each disease
DatasetID	✓	Datasets (DatasetID)	The ID defined for each dataset
FeatureID	✓	Features (FeatureID)	The ID defined for each feature
FeatureRank			The reported ranked of the feature

Based on these tables, we prepared various reports to extract important information from them. The description of the most important reports is shown in Table 16. As there are many reports extracted from this database, the description of other reports is available from our web application help section.

**Table 16:** Some of the most important reports and their descriptions.

Report name	Report description
Frequency of winning machine learning methods	This report shows how popular/successful a machine learning technique is. For each machine learning technique, it reports on the total number of papers that have used it in their analysis, and how many times it outperformed the other techniques.
Important features reported for a specific disease in a specific country	This table gives a detailed list of what features are collected in each country for each disease. Moreover, one can see the number of papers that have emphasized the importance of each feature, as well as the mean of ranks given to that feature as a proxy of feature importance in that country.
Comparison of machine learning methods in each dataset	Usually, each paper reports the results of applying multiple machine learning methods on a dataset. This table allows us to compare how these algorithms perform on a dataset. It reports the paper title, the dataset that it has used, and the difference between the accuracy of two selected methods.
Important features in each disease	This report lists the set of features that are reported to be important for each heart disease. First, a disease needs to be selected from the drop-down menu. The resulting table will show the feature name, the number of papers that reported the feature to be important for the selected disease, and the mean of the reported ranks for that feature. The smaller the rank, the more important the feature is.
Papers vs. Specific feature category	Feature categories represent the set of features that are obtained from the same resources. For example, ECG category represents the set of features that are obtained from electrocardiography. For each feature category, this table reports the paper titles and the accuracy they have achieved.
Number of papers using a specific algorithm by year	For a given machine learning method, this table reports the number of papers using that method per year, the title of publication with the best performance and the highest accuracy achieved.

## Data Records

**Fig. 1:**
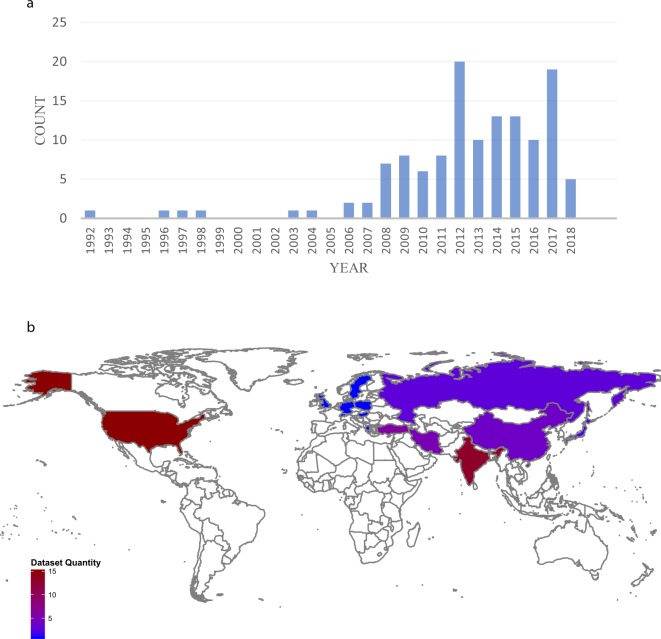
Source of our dataset and its distribution worldwide. (**a)** The number of sources included in the database by year of publication. (**b)** The datasets’ distribution in different countries. It is clear that most datasets were collected in the USA. Then, India, China, Turkey, and Iran have more datasets.

We chose to index papers related to CAD detection using machine learning and data mining approaches that are published between 1992 and 2018. These criteria result in 126 papers (See Fig. 1a) in which 490 authors contributed to these papers. These papers studied 68 different datasets with almost more than 360 distinct features collected in 18 countries from Asia, Europe, and America (See Fig. 1b). In these papers, 140 different machine learning or data mining techniques were applied to diagnose CAD. We extracted the data from these articles. They are available at www.cadataset.com and within figshare^51^ at 10.6084/m9.figshare.8092514.

**Fig. 2:**
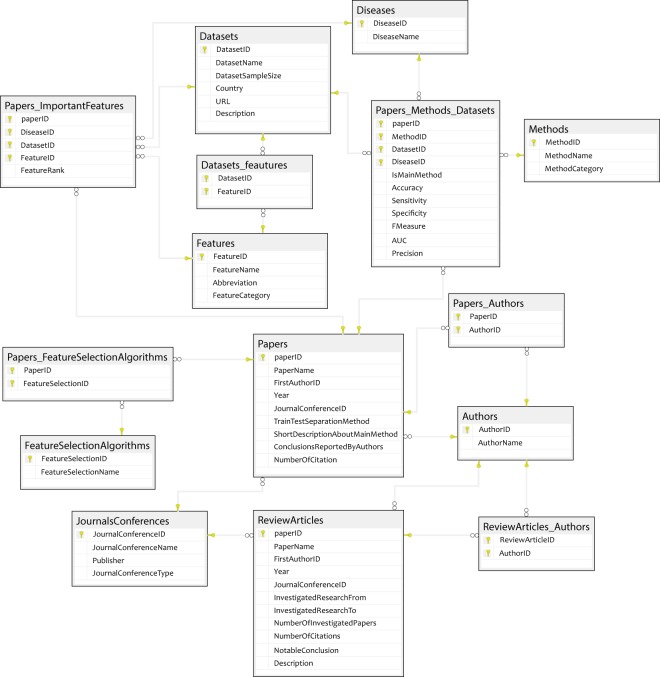
Structure of the database (The relationships between tables). The key icons in the tables show the primary keys of those tables, and the key icons in the relationships between tables show the source tables of foreign keys in the tables.

These data were saved in tables shown in Fig. 2. This figure shows the tables, their fields, the primary keys, and corresponding relationships between tables. In these tables, we saved the research and their properties. We tried to design the dataset with minimum redundancy in the saved data using the methods proposed by Silberschatz *et al*.^52^. For more information about tables and their fields, please refer to Tables 1–15.

This database has been presented in 3 different formats: comma-separated values (csv), Mysql script file (.sql), SqlServer script file (.sql). To use the csv file, the file should be opened in excel or imported to the database. To use Sqlserver files, an empty database in SQL server management studio named “cadataset” should be created and then run the script file (.sql file). To use Mysql files, an empty database in MySql named “cadataset” should be created and then run the script in the database. Users can download the data and SQL codes from our designed website at www.cadataset.com. The whole database underlying the CAD DATASET website was uploaded to figshare^51^.

Missing values were included as empty cells if they were not foreign keys. If they were, we defined a new record for them in the source table. For example, as datasetID in table Papers_ Datasets_Diseases_Methods is a foreign key, if the used dataset in a paper is not mentioned, we cannot let this field exist as a null field. Therefore, we defined a record in the table datasets as “not mentioned” and used its ID in the tables in which datasetID was a foreign key.

## Technical Validation

The retrieved articles (254 articles in total) of our paper were reviewed by 7 authors. To collect the dataset, we used the following keywords in Google Scholar:

(LAD OR LCX OR RCA OR CAD OR “Coronary artery disease” OR “Atherosclerotic heart disease” OR “Atherosclerotic vascular disease” OR “Coronary heart disease”) AND (disease OR failure OR diagnosis OR prognosis OR treatment) AND (“machine learning” OR “data mining” OR “machine intelligent” OR classification OR clustering).

The validity of our research is investigated according to the two following factors: first, the relevance of an article to the topic must be confirmed with at least five out of seven authors of the current paper. Meanwhile, if the article was published in an unreliable journal or conference, it was rejected. As each particular data value in the database has primary resources, users can evaluate the validity of information in the database. Finally, 125 papers were selected as our main articles and saved in our database. Our extracted results are designed according to the aggregation of the results of 125 articles; therefore, a low probability of an error in one study can influence the overall extracted results of our research. Second, the extracted results were confirmed by the six outstanding cardiologists. They validated all of the final results achieved in this research. They investigated all the text of the research, extracted plots, and tables and figures precisely and confirmed the results according to their knowledge and experiences.

## Usage Notes

### Web application

**Fig. 3:**
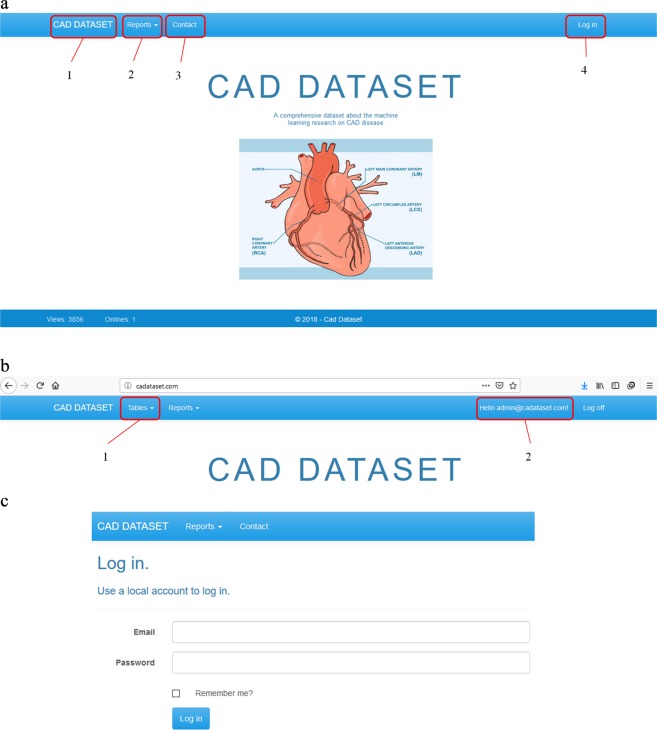
The front page of our web application in different modes. (**a**) The front page of our web application before login. There are four options. The first is a link to the home page. The second shows the list of reports that all users can see. The third is contact information, and finally, the fourth is used to log in/off to the system. (**b)** The front page after login; two more options appear. The first shows the list of tables, and the second shows the email address of the logged in user. (**c)** The login page. Currently, only the administrator can login to add, edit and remove data and reports. Other users do not need to login.

**Fig. 4:**
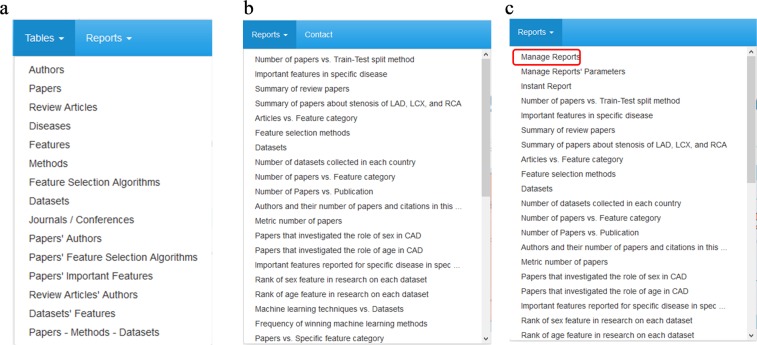
List of tables and reports. (**a)** A screenshot from the list of tables. Guests cannot see the list of tables (**b)** A screenshot from the list of reports (**c**) A screenshot from the list of reports for the administrator. Please note there is another option in the list that the administrator can use to manage the reports.

**Fig. 5:**
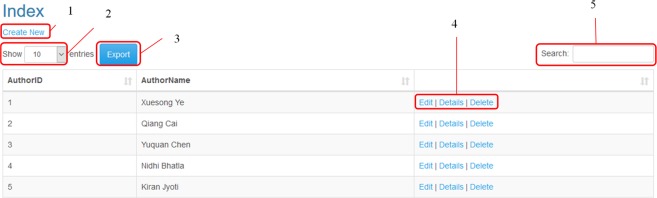
The facility prepared for an administrator to add, edit, and remove the data. The first option is used to add a new record to the table. The second option is used to determine the number of rows shown on a page. The third option can be used to export the table to the CSV file format. The fourth option can be used to edit or delete the specific record, and finally, the fifth option can be used to search the table.

**Fig. 6:**
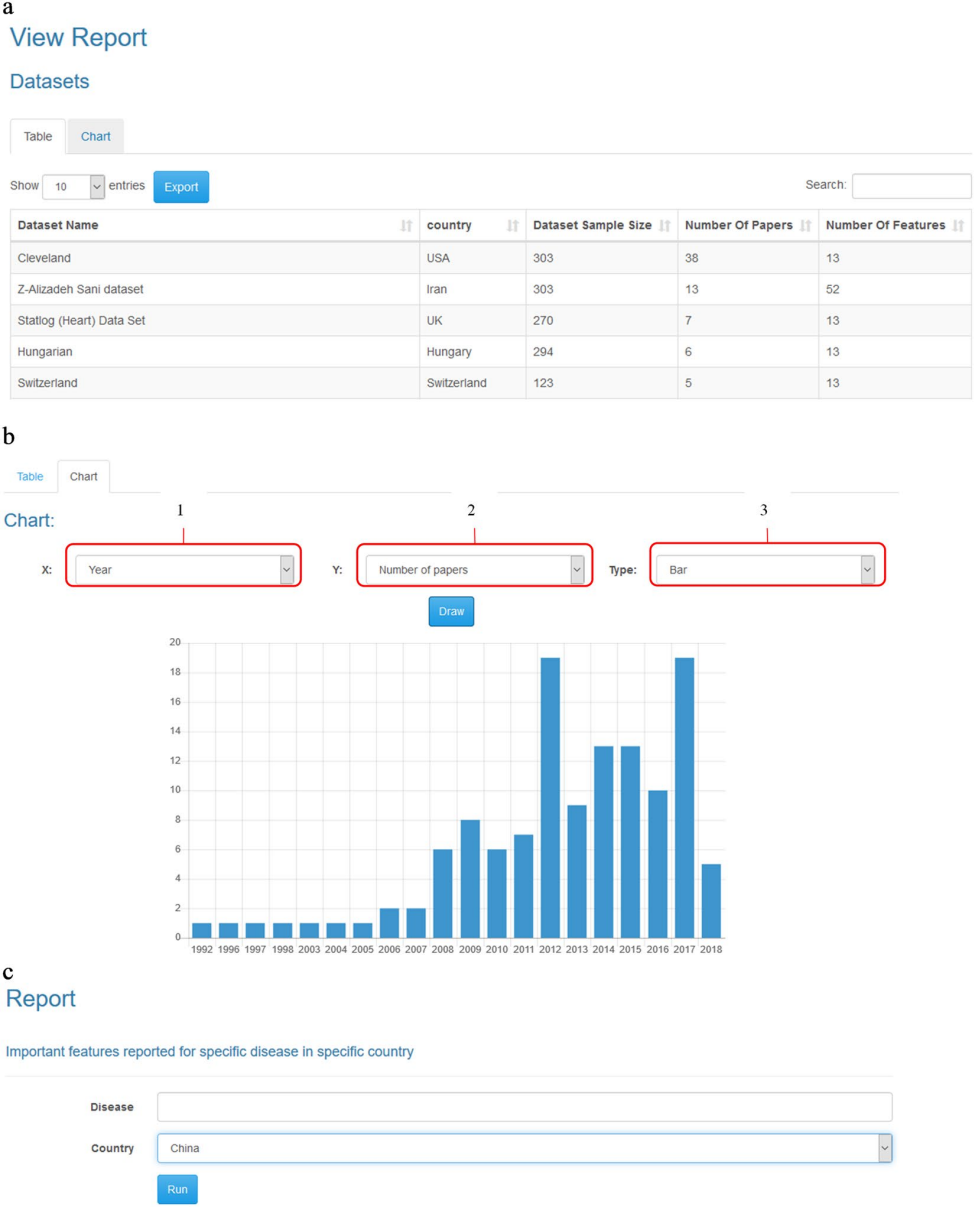
Reports. (**a)** Shows the output of a report as a table. (**b)** Shows output as a chart. The first and the second options in this form determine the horizontal and vertical axes, respectively. (**c)** In some reports, filters must be applied to data. For example, in this report, we need to specify the disease and country in which we are interested to see the most important features reported.

To use this database, we designed a web application that allows the project administrator to add, remove or edit the records of tables. As shown in Fig. 3, the users can access the facilities according to their permissions. As shown in Fig. 4, the administrator can manipulate tables and reports. However, other users can only see the report results. The facilities prepared for the administrator and other users are shown in Figs 5 and 6, respectively. Moreover, a video clip was created to explain the usage of this tool. It can be viewed on http://cadataset.com/help.

The database was last updated on 1/10/2018. Some records may change after this time, for example, the number of citations of an article, number of articles published by a publisher, and number of articles published by a researcher. For this purpose, this database is designed such that it can be updated easily. Once the database is updated, all the reports will be updated automatically. The database is also designed to allow checking the compatibility of new information such as the author’s name and journal name with previous information.

## Data Availability

The extracted data from the investigated articles were stored in an SQL database. See the database structure section for details of database tables. A web application was developed as an interface to interact with this dataset to extract the statistics of the saved data. The server-side application was developed using Microsoft SQL Server Express 2016 and ASP.NET MVC 5. The user interface uses the Bootstrap framework in addition to customized JavaScript libraries for plots, tables, and menus. SQL database and web application source code are available at www.cadataset.com and within figshare^51^. While the administrator of the website can modify previous records and add new data to the system, its reports are available to all users. It is possible for users to request new reports to be added. The reports can be exported to CSV format. In addition, the results can be plotted and summarized in a simple figure as well. Our website is compatible with all popular modern web browsers (tested on Mozilla Firefox ver. 63, Microsoft Internet Explorer ver. 11, and Google Chrome ver. 70).
